# Free Riders and Pious Sons – Why Science Research Remains Obligatory

**DOI:** 10.1111/j.1467-8519.2008.00648.x

**Published:** 2008-04-25

**Authors:** Sarah Chan, John Harris

**Affiliations:** 1 Ethics and Innovation, School of Law, University of Manchester; 2 Ethics and Innovation at the School of Law, University of Manchester

**Keywords:** duty to research

## Abstract

John Harris has previously proposed that there is a moral duty to participate in scientific research. This concept has recently been challenged by Iain Brassington, who asserts that the principles cited by Harris in support of the duty to research fail to establish its existence. In this paper we address these criticisms and provide new arguments for the existence of a moral obligation to research participation. This obligation, we argue, arises from two separate but related principles. The principle of fairness obliges us to support the social institutions which sustain us, of which research is one; while the principle of beneficence, or the duty of rescue, imposes upon us a duty to prevent harm to others, including by supporting potentially beneficial, even life-saving research. We argue that both these lines of argument support the duty to research, and explore further aspects of this duty, such as to whom it is owed and how it might be discharged.

## INTRODUCTION

In a recent issue of this journal,^1^ Iain Brassington has offered a commentary on a paper by John Harris entitled ‘Scientific Research is a Moral Duty’^2^ which argued, from a number of standpoints, that there is a moral obligation to participate in scientific research. Brassington, in his piece, purports to refute the arguments for any such duty to pursue, support and participate in scientific research.

The questions of whether or not there is a duty to support and participate in scientific research, and also of whether or not research is one of the highest social and political priorities, are of vital concern in bioethics. Although as we will demonstrate, Brassington's critique is ultimately misguided, it is nevertheless welcome, not least because it offers an opportunity to revisit this important issue.

## TWO PRINCIPLES

Part of the reason Brassington fails in his attempts at refuting the paper's arguments lies in the way he interprets, or rather misinterprets the arguments in question. He states that ‘in a nutshell, Harris’ claim is that obligations to participate in and support ongoing medical research derive from the benefits of past research that we currently enjoy’.^3^ This, however, is the wrong ‘nutshell’.

In fact, Harris cites two principles in support of the obligation towards research: firstly, that of ‘do no harm’ (and its weaker corollary, the duty of beneficence); and secondly, the principle of fairness. Brassington reconstructs these into three arguments: the ‘free rider argument’, the ‘argument from filial piety’ and the ‘argument from rescue’. Of these, the first derives from the principle of fairness; the second may be a misconstrued version of the same principle, but misconstrued in such a way that renders many of the arguments raised against it irrelevant. The ‘argument from rescue’ relates to the duty of beneficence and harm-avoidance, but fairness should also play a part in its application. When interpreted in this fashion, as we argue it should be, the argument from rescue supports (rather than contradicts, as Brassington would have it) the free rider argument. However, let us deal with his objections in turn.

## THE FREE RIDER ARGUMENT

The ‘free rider argument’, briefly summarized, goes as follows: if you benefit from an institution or practice, such as the ongoing institution of scientific research, and accept the benefits that derive from that institution, then you have, *in fairness*, a reason to support the existence of that institution or participate in that practice. This applies not just to research, but to multifarious aspects of modern society such as the existence of a welfare system, public education and health care. It would be unfair to accept willingly the benefits of a social institution such as the NHS or scientific research without also being prepared to support and, where necessary (and reasonable given the balance of burden and benefit) participate in that institution.^4^

The first counter-argument that Brassington raises against this claim is that non-participants in research are not, in fact, free riders: it is untrue, he contends, that they ‘accept the benefits of scientific research … without making any contribution in return’ because ‘one pays in some way for just about every medical benefit that one might enjoy’.^5^ In other words, although it may be unfair to act as a free rider, those who fail to contribute to research by participating are not free riders because they pay for the benefits they garner: through tax or medical insurance if not directly; and therefore there is no unfairness involved.

The applicability of this as a rebuttal of the original argument is complicated and requires consideration on a number of counts. It is true that we pay in some form and to some degree for the benefits of medical research; true also that if I pay taxes that go towards research, in a sense I am supporting that research, as by paying taxes I support anything that those taxes are used to support. So it is not the case that our alleged ‘free riders’ make *no* contribution to research. But does this invalidate the assertion that they might, out of fairness, still owe some obligation to it?

The unfairness of being a free rider does not rest solely on the ride being entirely free, but on the injustice of reaping a benefit beyond that to which one ought to be entitled on the basis of one's contribution. If a ‘ride’ costs £10 and I contribute only 50p, my ride is not literally free but, nevertheless, by shirking part of the necessary contribution I may be committing an injustice – as are those who refuse to contribute what is necessary to maintain the institution of research, even if they have paid something towards it in some form. The point is not whether *any* contribution is made, but whether a *reasonable or sufficient* contribution is made.

### What is a sufficient contribution?

Having established that the obligation from fairness to support research cannot necessarily be discharged merely by the making of *some* contribution (in this case the payment of a certain sum of taxes) but that there is also a criterion of sufficiency, we must now consider the question of what would constitute a sufficient contribution. Brassington himself brings up and dismisses the notion of sufficiency as a requirement to satisfy fairness: ‘If my insurance contributions are insufficient to pay for my treatment … this makes no difference, since any contribution I might make individually to scientific progress would likely prove nugatory in terms of the effort that goes into important discoveries.’ This is false logic – the cost of one person's health care, even if significant at the individual level, is also negligible in terms of the entire NHS budget – but in any case misses the point of the sufficiency criterion as it relates to justice. A sufficient contribution from each person towards the upkeep of an institution does not mean enough to counterbalance that particular individual's benefit from, or use of, that institution, but means enough that across the entire breadth of everyone's contributions, the institution can be adequately maintained and provide the services that are both wanted and needed. It is not whether my insurance contributions are sufficient to pay for the entirety of my treatment, but whether they are sufficient to make up my fair share of what is required across the board to support the medical research from which I am benefiting.

### Money is not everything

The assertion that because we all pay tax or medical insurance fees we are not free riders in the context of supporting scientific research also fails on another ground. Money, although certainly crucial for research, is not the sole thing that enables research to occur; the availability of willing research subjects may also be a limiting factor, where human participation is required. If my tax payments suffice to cover my share of the total resource cost of research, including generating enough subjects to meet the needs of the research, well and good; but if money alone cannot provide the necessary participants (or for other, possibly misguided resasons the payment of research subjects is ruled out),^6^ then a contribution in more than just monetary terms may be required.

Thus our duty to research extends beyond tax and insurance payments in the dimension of the nature of the obligation as well as the amount. We may, if it is needed for research to continue, have an obligation to participate in research ourselves as well as pay money towards it in whatever form. That being the case, such an obligation cannot be discharged by mere monetary payment.^7^

These considerations of how the obligation to research might be discharged point to a further flaw in Brassington's argument, relating to the foundation of the duty to research. He states that as the research and treatment have been paid for (by whatever means) then ‘if there *is* a duty to contribute to that which benefits me, it is one that I have already discharged. There is no further duty to research.’^8^ But the duty to research is not discharged solely or simply by payment: it is grounded (at least in part) in the need for the research, in the moral reasons we have to pursue research and in the goods at which the research is aimed; and if the need remains unfulfilled, the duty remains in force.

By analogy, imagine a situation in which a swimmer is drowning at the beach. All the beach-goers have contributed to pay for a lifeguard, whose duty it is to rescue anyone in danger of drowning; but the lifeguard has most irresponsibly abandoned his post and is nowhere to be found. The mere fact that every beach-goer has paid towards maintaining the presence of a lifeguard does not excuse them from trying to save the drowning swimmer in the lifeguard's absence, or make it any less of a morally reprehensible act for them to stand by and watch the unfortunate victim expire for want of assistance. Or take as an example another institution of public benefit, the fire brigade. We all pay taxes which go towards supporting the existence of the fire service, but that does not relieve us of the obligation to attempt to put out fires we may see as passers-by when the fire brigade is not in evidence and the required dousing is within our capabilities.

In other words, you may pay someone to do your duty for you, but that in itself does not extinguish the duty or absolve you of all moral responsibility. If the payee is derelict in performing their duties, you may have a personal claim against them, but the moral obligation remains upon you – indeed the existence of that unquenchable obligation is probably the best reason you have for contributing to rescue on the first place. If the amount paid does not, for whatever reason, result in the obligation being fulfilled then it will remain in force despite the payment having been made. As applied to the duty to research, therefore, it cannot be said that paying taxes and medical insurance is necessarily enough to fulfil any existing obligations, or that having paid these entitles us to claim that ‘[t]here is no further duty to research’.

Now, it is true that the payment of an adequate sum might suffice to satisfy the duty to research from fairness *if* fairness were the only ground for the duty. But, as the original paper attempted to demonstrate, this is not the case: there are at least two major lines of argument which establish the duty to research and act in conjunction to support it and each other, these being the duty from fairness that has been termed above the ‘free rider argument’, and the duty of rescue. We shall explore the latter in more detail, but first let us address the remainder of Brassington's attempts upon the free rider argument.

### Free riders do commit injustice

The final criticism levelled at the free rider argument seems to return to the notion that non-participation in research is not free-riding as such. ‘If I decide not to pay what I ought for the provision of the benefits of research … then I have certainly wronged those around me. However, the nature of the wrong is not that I have been a free rider, it is that I have been a thief.’^9^ Although this is scarcely any better, Brassington argues that ‘there is no reason to believe that those who benefit from good things such as are provided by medical or other forms of research *are* thieves’ because, as per his arguments as discussed above, they pay taxes. This, however, as we have established, does not automatically absolve them of the duty to participate, nor does it necessarily completely constitute paying what one ought. He goes on to say that ‘[t]hey might think they have to pay too much – but this is beside the point: any rational person will seek to avoid having to pay much for anything, and isn't thereby rendered a bad person.’^10^ But this is precisely the nature of the injustice: persons avoiding having to pay what is required of them in money, time or personal contribution to justify the benefits they receive. Whether we call them free riders, thieves or something else, the argument stands. Moreover, two further points must be made about the rationality of free riding. The first is that if free riding undermines a social practice that is by hypothesis morally and practically important, then it is not rational. And while there are circumstances in which it might be rational to act unethically it can never be ethical so to do. And that is the point.

The core of Brassington's criticism is hence false, but he also makes some intriguing statements along the way regarding the injustice of free-riding (or alleged absence of injustice). These, although they contribute little to the final conclusion, are worth addressing as doing so may serve to strengthen and further illustrate the argument we are defending: that the requirements of justice impose a moral duty to support research.

According to Brassington's reconstruction of the free rider argument using the example of two housemates with differing levels of hygiene, ‘Jack has not wronged Meg by not cleaning the kitchen’ because ‘[s]he would have cleaned the kitchen anyway’,^11^ presumably in order to achieve the level of cleanliness required. This is not strictly true, however: she would not have had to do so if Jack had done his share. By such logic we could say that Jack would not wrong Meg by failing to pay his half of the rent, because under such circumstances she would most likely pay his share to avoid being evicted; but this is patently false! A free rider's disrepute may or may not stem from his injustice;^12^ but his moral failure certainly does. The assertion that ‘[n]either Jack nor Meg suffers a kitchen that is more dirty than they are willing to tolerate’^13^ may be true, but that is not the only moral wrong: Meg is being exploited as a result of Jack's failure to hold up his end of things. It might also be true in the event that Jack leaves the rent unpaid that ‘neither tenant suffers a flat more unpaid for, or a legal obligation more breached, than they are willing to tolerate’ but this ‘fact’ is hardly exculpatory in law or ethics.

The injustice is no better when it has multiple or community victims instead of just one, as in the example of immunization. Benefiting from herd immunity while avoiding the risk of being immunized oneself is not only free-riding but, contrary to Brassington's assertions, *does* wrong other people even if those people would have been immunized anyway. It does so firstly because the un-immunized individual, being more likely to contract the disease as a result of not being vaccinated, indirectly imposes the costs of her risk of illness on the rest of the individuals in society who must bear the tax burden of providing her additional health care; and secondly because not all individual cases of vaccination are effective in conferring immunity, and therefore by increasing the risk of disease within the herd, an un-vaccinated individual directly exposes others, who have done their bit by being vaccinated themselves, to harm.

This is similar to the case where an individual's particular contribution to research is ‘nugatory’: an ineffective vaccination may not itself promote the herd immunity that is of value to the community, but the collective effort (of which each individual vaccination is a part) is required to produce the communal benefit. Likewise a collective obligation to participate in research is required if we are to reap the benefits of research, and to shirk that obligation is a moral wrong against the members of our community.

## WHAT IS ‘FILIAL PIETY’?

We now turn to the second strand of Brassington's criticism, which he terms the ‘argument from filial piety’. He explains this using the example of Socrates in *The Crito*, who accepts his death sentence because it is his duty to submit to the lawful processes (no matter how unjust) of the society that raised him; and goes on to produce some further implausible illustrations of the AFP and how it fails. These however, although apt as demonstrations of the invalidity of the AFP, are completely wide of the mark in that they have little relevance to the manner in which the original argument was framed or, indeed, to its content.

‘A present person does not face obligations just because he would not be where he is today without certain past events.’^14^ This is entirely true, but is orthogonal rather than antithetical to the original argument, which was about duties derived from fairness and justice, not history. Indeed, we agree that a person is not obliged to support everything that has been a pre-condition of her existence. The obligation arising from fairness to support institutions that provide a benefit and from which one benefits does not remove the element of moral judgment as to which institutions we ought to support. Socrates did not have to support a corrupt system of law that convicted him and condemned him to death on trumped-up charges; I do not have to support the institution of marriage merely because I came into existence as a product of my parents’ marriage! One does not have to support *everything* which has contributed to whom one is today; this may be a version of the argument from filial piety but is unrelated to the fairness argument which was the central tenet of the original paper.

In fact, Brassington's identification of the AFP as an argument presented in support of the duty of research is mistaken or misdirected. Although it was noted that ‘[m]any of us would not be here’ were it not for the fruits of research, this was intended to illustrate the beneficial nature of research and the fact that we have benefited from it and will continue to do so. It is this that imposes the duty; not the mere fact that research has been a precondition of our current existence.

## THE DUTY OF RESCUE

The duty of rescue refers to our obligation neither to cause nor fail to prevent harm. It is probably worth restating the original formulation of this principle for the sake of clarity of argument: ‘[w]here our actions will, or may probably prevent serious harm then if we can reasonably (given the balance of risk and burden to ourselves and benefit to others) we clearly should act because to fail to do so is to accept responsibility for the harm that occurs’.^15^ Failing to prevent harm is as effective a way of ensuring that harm occurs, and hence as morally reprehensible, as doing harm directly.^16^

### Many forms of rescue

Brassington asserts that the application of this principle to construct a duty to research ‘misrepresents affairs’ … ‘even if engaging in research *is* a form of rescue, it is not the only form of rescue, and we might still have a moral reason to pursue the other forms ahead of research’.^17^ The argument, however, was not that we have an obligation to perform *only* this type of rescue or even to prioritize this form of rescue over particular other forms, but that there does exist an obligation to this type of rescue, as to any type of rescue: there is an obligation to support all sorts of public goods.

Of course we may have moral reasons to pursue other types of rescue; we may have competing obligations, in which case the rational course of action is to prioritize. The existence of competing obligations or alternative forms of rescue, even the fact that under some circumstances these may take moral precedence, does not negate the existence of the obligation to support and participate in the particular form of rescue that is research participation.

Thus the examples of other forms of rescue that might save lives, such as providing resources in the form of food or health care, while valid as forms of rescue and therefore perhaps moral obligations in themselves, do not invalidate research as another form of rescue and an obligation. Further, although these obligations may compete for priority, they are not necessarily mutually exclusive: perhaps it is a ‘more pressing duty to relieve [immediate] suffering’ than to participate in research that will prevent future suffering, but we may be able to do both; and if we can, we should. If I am on my way to an appointment as a research participant when I see a child drowning in a pond, it is clearly the rational course of action to default on or delay that day's research in order to fulfil the more pressing obligation of rescuing the child; but that does not mean that I am free of the obligation to the more distant potential rescue, or that the next day, when there is no drowning child to divert my time, I should not return to participate in the research as originally intended.

### Prioritizing rescue: present versus future

To be sure, resources – time included – are never in such bountiful supply as to allow us to perform every rescue and fulfil every obligation. If they were, then (as Brassington concedes) the application of the duty of rescue to create an unimpeded obligation to research would be clear and simple. But given that they are not, can the obligation to research ever be a duty worth prioritizing? Can rational prioritization still lead us to conclude that people ought to participate in research as a moral duty?

As argued above, we believe that it can because even under circumstances of limited resources, participation in research and other modes of rescue are not always, or even often, in direct conflict. We wish, however, to address the question of prioritizing obligations to rescue with reference to the arguments about the relative importance of present versus future rescue. According to Brassington, research is directed at saving future people and since ‘the future always comes at a discount’, it will always be more imperative to engage in activities that save people in the present, such as donating money to provide food and health care. He illustrates this by saying: ‘Given the choice between saving either current-me or future-me from pain, I would hope that bystanders save current-me’, not only because of the possibility that future pain may not come about after all, but because ‘current pain hurts, and future pain doesn't – I can distance myself from it’.

There are a number of difficulties with this line of argument. First of all, research may save not only future people but also current people at a time in the future. Therefore, although it may be problematic from a particular perspective to establish a present duty towards people who do not yet exist, the duty towards research is also a duty of rescue towards existing people. Does the fact that this rescue will be effected in years to come rather than immediately make it less of an obligation, as Brassington would have us believe? Why *should* the future come at a discount?^18^

John Broome^19^ has addressed this question and offers the following possible justification as to why future lifesaving rescues may be worth less than present ones:^20^

Lifesaving may not be a constant-well-being commodity. Undoubtedly, saving some people's lives adds more well-being to the world than saving other people's. Saving a twenty-year old with a long and happy future ahead of her adds more well-being than saving a ninety-year-old with little left to look forward to. We may expect that, by and large, a society will first direct its resources to saving the people with most well-being to gain. As it progresses in its ability to save lives, it will start to save people with less and less to gain … Therefore the more lifesaving is deferred to the future, the less well-being it will produce on average at the margin … Eventually … there will come a point where the power price of future lifesaving is matched by its lower benefit in terms of well-being. At that point, future lifesaving is on average genuinely less valuable than present lifesaving. Lifesaving should then be discounted.

Broome is mistaken in treating life as a commodity and his conception of what life-saving means commodifies life comprehensively. The prohibition against commodifying life is usually attributed to Immanuel Kant^21^ and is understood as the idea that life is commodified when persons are treated not as ends in themselves but as means to the ends of others, as mere means. There are two ways of commodifying life. The first, and most usual, is where human life, or more precisely the life of a person, is treated to some extent as capable of commodification. An example is the prohibition on commodification in the Oviedo Convention which, in purporting to outlaw the sale of body parts, does so because it sees such sale as treating the human being as a means to the ends of others.^22^ It is in fact doubtful whether the sale of body parts or bodily services is against Kant's famous dictum because such sales are compatible with treating the human person also as an end in him or herself. Broome however goes the whole hog and takes the second way, identifying the *value* of the life of a person as the quantum of well-being that life adds to the world. For Broome the reason to save a life is to maximize the amount of well being such an action adds to the world. This is seeing the value of life exclusively as a commodity, as the amount of well-being it contains and the value of saving a life as the quantum of well-being that life-saving adds to the world.

But well-being, or indeed welfare, is not an end in itself, it is an instrumental good, not a good that benefits the world in proportion to the amount of it there is floating about, but rather a good that benefits the individual person whose being is well (or otherwise). Well-being is the welfare of a being, not a quantum of abstract goodness to be maximized. Concern for or promotion of well-being or welfare is then a state of being of a person, not simply a state of the world, it complements an individual's autonomy in that it provides the conditions in which autonomy can flourish and lives be given their own unique meaning. Concern for or protection or promotion of well-being or welfare ceases to be legitimate at the point at which, so far from being good for an individual, productive of their autonomy, so far from enabling the individual to create her own life, it operates to frustrate the individual's own attempts to create her own life for herself. Well-being and welfare thus conceived has a point, as does concern for the welfare of others; it is not simply a good in itself. We need welfare, broadly conceived in terms of health, freedom from pain, mobility, shelter, nourishment and so on because these things create the conditions which not only maximize autonomy but also give autonomy the minimum scope needed for operation. In this way welfare is *liberating*, it is what we need to be able to pursue our lives not only to our best advantage but also in our own way.^23^ The value of a life is a value overwhelmingly to the individual whose life it is, and to the person, the loss of their life is the loss of everything, not simply of something or some things. This is why, as Harris has argued on a number of occasions including against other ideas of John Broome, the value of a life is not proportional to the amount of good or well-being or welfare it ‘contains’ nor to the amount of lifetime enjoyed or in prospect for the individual whose life it is. For that individual, however well (or ill) their being or however long or short their life or lifetime in prospect, it is the loss of everything. That is why it is not simply wrong-headed but wrongful (full of wrong) to value lives differentially according to quality or quantity of life.

If the millionaire and the pauper both lose all they have, on one way of thinking about the loss, each has suffered the same degree of loss: each has lost everything. On another, each has suffered a different quantum, of loss measured by the total sum lost. There is no straightforward way of reconciling these different approaches to the assessment of loss. If we are searching for an equitable approach to loss, it is not obvious that we should devote resources allocated to loss minimization to ensuring that the millionaire is protected rather than the pauper. The same is true of health gain or indeed of well-being or welfare. Even if it is agreed that resources devoted to welfare or health care are resources devoted to minimizing the loss of health or welfare or well-being, it could not be demonstrated that the person who stands to lose more well-being or more life years if they die prematurely, stands to suffer a *greater loss* than the person who has less well-being or life expectancy.

If you and I are competitors for rescue or life-saving care and I have already or will have after the rescue more well-being or better welfare than you, but both of us will receive something that is significant and important to us, automatically preferring to satisfy my needs rather than yours, seems unfair. Why should my life be judged more worth saving because I'm more healthy or happy or have greater well-being, rather than more intelligent, say, or more useful? Arguments can (and have) been made on both sides, but to define need, for example, in terms of capacity to benefit and then argue that the greater the wellbeing deliverable by rescue, the greater the need for rescue (or the greater is the person's interest in receiving rescue,) is just to beg the crucial question.^24^

The future *may* come at a discount for some commodities; but lives and lifesaving are not among them.

Let us now address the question of whether we can have duties to non-existent people, future people, which we noted causes problems on some views of obligation or rights.

We believe this problem about duties to future non-existent people is largely illusory. While future people (people who as yet do not exist) have no rights and do not exist to make claims upon us now, it does not follow that we cannot harm them and therefore that they are not covered by all our person affecting duties including our duty not to harm others. Consider, if we put a slow acting poison into the water supply, a poison that will not become active for two hundred years, it will kill no one presently alive but everyone who drinks the water in two hundred years time. Such an action would not be harmless. While we cannot *identify* in the sense of *name* those who will die, we can identify them in another sense. They are all those who will derive their water supply from x,y and z reservoirs in two hundred years time. Since our action will affect persons, future persons, it is part of person-affecting morality; and since it will cause a particularly harmful form of harm, namely death, it is covered by our duty not to harm others and our duty not to kill others. This sort of future harm is not discountable.

What is true of harms is also true of benefits; the two are the Janus faces of the duties we have to others.^25^ Just as we have the same reason not to cause future harms as we do not to cause present harms, we have the same reason to confer future benefits as we do to confer present ones. A duty to benefit future people by engaging in research that will save their lives is no less important than a duty to benefit current people.

Now let's consider the case in which the future people do already exist but are different people.

Intuitively it seems correct that a duty to rescue X today is more pressing than one to rescue Y in a year's time. But it seems likely that this is due to the probabilistic intuition that during the intervening year, something else may occur to render our duty to rescue Y unnecessary or irrelevant. If we could say with 100% certainty that without our intervention, X and Y would both suffer equal injury but at different times, it is hard to see why our obligation to X is greater than that to Y. The reasoning that one can distance oneself from future suffering only applies in the absence of forethought: future pain *will* hurt, in the future, and choosing to avoid the present pain does not make the overall suffering any less. In fact one might argue that, if one is not cushioned by the comfortable lack of foresight by which Brassington seems to be protected, it would be better to undergo the pain now and hence avoid the mental torment caused by living in dread of the pain to come. In the case of saving lives, matters may be slightly different. It is clear that future-me will not exist if current-me is not rescued: the dereliction of one duty precludes the exercise of the other. This is not, however, the case when the present and future duties are owed to different parties.

The ethics of discounting the future where different people are to receive the benefits of rescue is complicated by two further considerations. The first is easily dealt with. While it is true that we are, in some ways, constantly changing, can we say that Y-in-one-year's-time exists now in the person of Y? If and insofar as this is right, the problem of my trade-off between present and future rescue from harm may reduce to the problem of whether saving X now or Y in the future have different priorities. We do not believe so because even in the unlikely event that ‘me’ in one (or even twenty) years time is not really me, there will be enough psychological continuity between the two of us to make it rational for me now to have a strong interest in what happens to me modified in twenty years.^26^

More significantly, if we opt to rescue X instead of Y, Y still gets an extra year of life. On a purely numbers basis, with no way of determining whose life is of greater ‘benefit’, this might make it better to rescue X. However, we would need theories about how the value of a life is varied by life expectancy or lifetime lived, not to mention cost of rescue and many other features, before this conundrum could be finally resolved.^27^

Therefore, we would argue, the intuitive appeal of this line of argument comes from underlying assumptions about the likelihood of whether the future rescue will be either necessary or effected, in comparison to the relative certitude and necessity of saving someone in need today. True, it seems that giving £5 towards research that may or may not save lives at some unspecified point in the future is less worthwhile than giving £5 that will provide life-saving treatment tomorrow; but it cannot be the case that a guaranteed and necessary rescue tomorrow outweighs a guaranteed and necessary rescue in two days’ time, merely because the latter is further in the future. If that were the case, surely we would be obliged to seek out those charities that were closest to home for our life-saving donations (or even give money to the homeless and suffering directly) rather than giving money to foreign aid organisations where the benefits of our donation might take days, weeks or even months to percolate.

Now it may well be the case that the relative probability of the research to which one contributes producing life-saving results in the future is lower than the probability that £5 given to Oxfam will save a life somewhere in the nearer future. But if we are going to attempt a calculus of likely benefit, we must also take into account that the life-saving interventions that may result from research, albeit at a lower probability, also have the potential to save *far more lives* than our meagre £5 will. An exhaustive analysis of the probability versus benefit equation would be nigh impossible to perform (and in the time taken to perform it, one could no doubt have participated in more than enough beneficial rescue activities to outweigh the difference!) but it cannot be said definitively that the benefit of a single, relatively likely current rescue outweighs the benefit of multiple less likely future rescues: the obligation towards research as a form of potential wide-ranging rescue persists. Somehow we must find time, resources and moral courage to do both rather than either one or the other.

Brassington would have it that such a calculus is irrelevant, that the lives of any number of future victims are discounted relative to the life of a current person at risk, because, as he claims, ‘[t]here is no duty to benefit the future’. This seems a dubious proposition, however. If you know a child is likely to drown tomorrow if you don't put a fence around your swimming pool, you have a duty to erect the fence even though the harm caused is in the future. The calculus of probability and benefit may affect the manner of our choosing between two courses of rescue, but the choice of present rescue over future cannot be justified by denying that there is any obligation to future rescue at all.

In any case, such distinctions are not especially relevant in practical terms, most obviously because people seldom face an either/or choice between research and other forms of rescue: research participation may not render a person less likely to contribute money to Oxfam nor vice versa. One thing, however, is clear: the obligation to participate in research as a form of rescue is not diminished merely because the rescue is in the future. When we set up a health care system or build a hospital we are investing in future rescue. It can hardly be the case that there is no powerful moral reason to build hospitals.

## DO THE DUTIES FROM FAIRNESS AND RESCUE CONFLICT?

We have thus far established that the objections raised by Brassington do not stand to invalidate the originally proposed obligation to participate in research: such an obligation can be asserted on the basis of both the principle of fairness, being the duty to contribute to what sustains us, and the principle of beneficence – the duty of rescue. One final objection remains to be addressed: Brassington argues that the joint application of both these principles is self-contradictory, that each negates the other. As he would have it, ‘a person who is rescued is … the excelsior of free riders’, since the rescuer ‘receives nothing … in return’ nor should expect to do so: ‘Rescuers have a duty to treat the rescued as *unproblematic* free riders’.

One might argue that it is not the case that the rescuer receives nothing in return: how many people are given the chance to perform the heroic deed of saving a life? It is, however, true that the duty of rescue entails a strong assumption that the rescue is not carried out primarily (or even partly) in expectation of some sort of recompense. Even so, that does not mean that those who are rescued are acting as free riders. To state this is to misunderstand the nature of the obligation from fairness and the duty of rescue, erroneously narrowing its scope to govern only relations between individuals. The rescued individual will, in all probability, never have the chance to render an equivalent return service to his rescuer by saving the rescuer's life in turn; but this is not what the duty of fairness demands in this context.

To explain this, let us briefly recap the content of these twin duties, the duty to rescue and the duty of fairness. The duty of rescue stems from the moral obligation to prevent harm and benefit others; the duty arising from fairness is the duty to support those social practices and institutions from which we benefit and the duty of reciprocity, to return good for good.^28^ The rescued individual has indubitably benefited from having his life saved by the rescuer who, acting under the obligation of the duty of rescue, is owed no additional moral debt by the rescuee for doing so. The duty from fairness does not compel the rescuee to repay to his beneficiary in exact coin and amount the benefit he received, even ignoring the unlikeliness of him ever having the chance to do so. What it does do is place an obligation on him to support the practice from which he willingly benefited or from which he was happy to accept the benefit, as it places an obligation on *all individuals* who have so benefited or might so benefit: to play the rescuer in his turn should he ever be called upon to do so. The rescued individual only becomes a free rider if he fails to uphold the same duty of rescue that has saved his life, by failing to attempt a rescue that he could have effected; and in that case moral shame ought rightfully to attach to him in full measure, as it surely would to anyone who stands by in idleness when he could have saved a life.

Most of us will never be in a position to play either rescuer or rescuee in so dramatic a manner as pulling someone (or being the someone pulled) about to drown from the sea. We all benefit, however, from living in a society where we can hope to be rescued should we ever be in need, even if that situation of necessity never comes to pass; and thus it falls upon us all to support the duty of rescue and fulfil the obligations it imposes if we are ever called upon to rescue another. Moreover, we *are* in a position to assist the rescue of others by our contributions towards research and we will most likely be ‘rescued’ by, or at least benefit from, the fruits of such research – in fact, have probably already done so. Thus the duty of rescue and the obligation from fairness operate in a concordant, not contradictory fashion: one reinforces the other, and both reinforce our duties to support, through our participation, research that has benefited us and will benefit others.

## THE DUTY TO RESEARCH: CONCLUDING REMARKS

We have here defended the existence of a broad obligation to participate in research with reference to its basis in fairness and the duty of rescue, without exploring the content of the obligation in any great detail. A few additional remarks, then, as to how the duty to research might be discharged; what might suffice to discharge it; and whether the obligation is in any way an enforceable one.

### Discharging the obligation to research

To determine what might count towards discharging the obligation, we must examine the content of the duty a little more closely. The duty of rescue imposes an obligation to prevent harm to others where reasonably possible: for example, to render aid to a swimmer whom one sees drowning. What matters for the discharge of this duty is that the rescue *is* effected, the swimmer *is* somehow saved. If that requires one person to attempt the rescue rather than every single beach-goer jostling and getting in each others’ way, so be it; one person's rescue can count for the obligation of all.

In this sense the justification for the obligation is not deontological but consequentialist: it is not the doing of duties, the attempting of rescues, that is morally right, but the beneficial outcome, the possibility of rescue itself, that creates the moral obligation to pursue it. It matters not whose contribution provides the necessary impetus to see the rescue out: if all are willing, the obligation may be discharged somehow. And if some people of morally upright character are always effecting rescues or fulfilling obligations on behalf of all? Why, then it comes back to the issue of fairness: moral indigents should not be free riders at the expense of those with a sharper conscience.

There may be people who are genuinely unable to contribute, or undergo greater risks in doing so – such as in our example above, a bystander who cannot swim. What does the moral obligation demand from these individuals; indeed, what can it fairly demand? The duty must be proportional to the risks and burdens of discharging it: fairness demands an equalization of risks and benefits in proportion. Income tax, at least in theory, works in precisely this manner. This is the corollary of the principle of how much is sufficient for each person to contribute: enough that the obligation is fulfilled collectively, *and* with each individual sharing the burden of contributing proportional to his own burden in doing so. The non-swimmer may find alternative ways of discharging social obligations and can pursue these if he is more suited to doing so.

In the case of research, then, if there is no reason to pay more tax or to be a research subject because the ‘job is done’, you have no obligation to do more. But if the job is not being done – if there is more that research could do to save others – the mere fact of having made some contribution does not absolve you of the responsibility to make a further contribution. In other words, if the obligation is to do a particular job, to bring about a beneficial outcome, and you do not do enough for that, you are responsible. The obligation persists in this case because of the failure to prevent harm: the duty of beneficence or harm-prevention is fulfilled only when the harm is prevented or the good is accomplished, not simply by attempts to do so.

### Where does research end?

An obvious difficulty in establishing the extent of the obligation to research is the open-ended nature of research: when can we say, with respect to research, that ‘the job is done’? What would constitute a reasonable obligation to research? This is not easily answered, certainly not within the scope of the remainder of our discussion. What can be said, however, is that most moral claims of this type are open-ended, and the fact that an obligation is unquantifiable does not negate its existence. How much money ‘should’ you give to charity or to good causes, how hard should you work to discharge your obligation to your employer? The absence of a definable answer to this question does not make giving to charity or doing a fair day's work any less of a moral good; neither does the problem of how much research is enough invalidate the obligation to pursue it.

### Research as an enforceable obligation? Shifting the paradigm of participation

There are two ways to understand the obligation to research: 1) a collective obligation that falls upon us jointly and severally, to make provision for the fulfilment of the obligation through the social apparatus that exists to do this; 2) an individual obligation that falls upon each of us. Fulfilment of the obligation may require something of us in both regards: individually by contributing (time, resources, participation) to the system, and collectively, such as by allocating taxes to fund the system and also by ensuring sufficient human resources to sustain it.^29^

How, then, should the last be achieved? For most public institutions, it is usually on a semi-voluntary basis: people are offered incentives in the form of salaries to work as researchers, health care professionals, teachers and so forth. It is also occasionally discharged in a coercive manner where required; jury duty and military conscription are two notable examples. In fact, we conscript people to participate in many public goods: enforced wearing of seatbelts, speed limits and compulsory education are all ways of compelling people to promote public goods.

It is not our intention to advocate unreservedly a system of conscription for research participation. Conscription is one way of universalizing participation, but not necessarily the only (or the best) way. If there are enough volunteers to sustain research, there is no need to conscript more; if people voluntarily support public institutions through donation, there is no need to levy taxes. The original purpose of venturing into this fray, though, was not to justify conscription to research participation, but to rebut the pervasive presumption that people would not, should not participate in research unless it offered some direct benefit to them or they were coerced in some way.

If there is an obligation to participate in research, we may reframe the notion of participation. Instead of a special endeavour above and beyond the call of normal social obligation, requiring extravagant safeguards even where actual risks are minimal and necessitating a degree of consent far higher than that required for most activities, we should regard research participation as something in which people have both a moral and a personal interest even where there is no immediate direct benefit, and presume accordingly that most of our fellow humans are of good moral character and would choose to do their duty unless there was an overriding reason to avoid doing so. This does not in itself justify conscription in any or all forms: there might be better means of producing the necessary result. Payment for participation might be one; perhaps persuasion and pointing out where our moral obligations lie is all that is required to convince people of their duties. We hope that in this paper we have achieved some small step towards doing so.

**Sarah Chan** is a Research Fellow in Bioethics and Law at the Institute for Science, Ethics and Innovation, School of Law, University of Manchester.

**John Harris** is Lord Alliance Professor of Bioethics and Director of the Institute for Science, Ethics and Innovation at the School of Law, University of Manchester. His most recent book, *Enhancing Evolution*, was published by Princeton University Press in 2007.

## Footnotes

1 I. Brassington. John Harris' argument for a duty to research. *Bioethics* 2007; 21(3): 160–168.

2 John Harris. Scientific Research is a Moral Duty. *J Med Ethics* Vol. 31. No. 4. April 2005. 242–248. 3 Ibid., p. 160.

3 *Ibid.*, p. 160.

4 For example by permitting medical students to be present at and participate in examinations as part of their training.

5 Brassington, *op. cit.* note 1: 161.

6 Harris argues that non payment is in fact misguided in *op. cit.* note 2: 242–248.

7 We ignore the possibility of paying others to do our turn as research subjects, not least because this takes ‘payment’ far beyond Brassington's minimal supposed contribution via normal taxation.

8 Brassington, *op. cit.* note 1: 162.

9 *Ibid*.

10 *Ibid*.: 163.

11 *Ibid.*: 162.

12 Although we feel strongly that it might do so.

13 *Ibid*.: 163.

14 *Ibid.*, p. 164.

15 J. Harris. *op. cit.* note 2: 242.

16 John Harris. 1980. *Violence and Responsibility*. London: Routledge and Kegan Paul.

17 *Ibid.*: 165.

18 See Derek Parfit. 1984. *Reasons and Persons*. Oxford: Clarendon Press: 356 ff; John Broome. Discounting The Future. *Philos & Public Aff.* 1994; 23(2): 128–156.

19 Here the argument follows lines developed in: M. Quigley & J. Harris. Personal or Public Health? In *International Public Health Policy and Ethics*. M. Boylan, ed. Springer, in press.

20 J. Broome, *op. cit.* note 18.

21 One version of the categorical imperative and the most pertinent for us is ‘[A]ct as to treat humanity, whether in thine own person or in that of any other, in every case as an end withal, never as means only.’ Kant, I., (1785) 2005, *Groundwork for the metaphysics of morals*, tr. Thomas Kingsmill Abbott, edited with revisions by Lara Denis. Peterborough, Ont.; Orchard Park, NY: Broadview Press.

22 Council of Europe *Convention for the Protection of Human Rights and Dignity of the Human Beings with regard to the Application of Biology and Medicine: Convention on Human Rights and Biomedicine.* Oviedo 4.IV.1997. Article 21 ‘The human body and its parts shall not, as such, give rise to financial gain.’

23 These ideas were elaborated in J. Harris. Consent and end of life decisions. *J Med Ethics* 2003; 29(1): 10–16.

24 See J. Harris. What the principal objective of the NHS should *really* be. *BMJ*. 1997; 314: 669–672.[Reprinted in Bill New, Ed. 1997. *Rationing: talk and action in health care* London, BMJ Publishing Group: 100–106.]

25 As John Harris argued in *Violence and Responsibility*, *op. cit.* note 16.

26 This is discussed by John Harris in *Enhancing Evolution* Princeton University Press, Princeton and Oxford 2007 Chapter IV.

27 See J. Harris.The Age-Indifference Principle and Equality. *CQHE*. 2005; 14(1): 93–99.

28 We say no more about reciprocity here and ignore the question of whether or not there might be a duty to render evil for evil, ‘an eye for an eye’, either in the case of individual reciprocity or in the case of institutional action.

29 Provided that the objective is accomplished, it does not matter how the obligation is discharged. For example, a health care system can be privately or publicly funded, it does not matter which as long as it fulfils the necessary role. The type of institution we choose to establish is irrelevant, provided it does what it is supposed to do.

